# Editorial: The pancreatic islet – a multifaceted hub of inter-cellular communication

**DOI:** 10.3389/fendo.2023.1182897

**Published:** 2023-05-17

**Authors:** Quan Zhang, Mark O. Huising, Gabriela Da Silva Xavier, Astrid C. Hauge-Evans

**Affiliations:** ^1^ Oxford Centre for Diabetes, Endocrinology & Metabolism, Radcliffe Department of Medicine, University of Oxford, Churchill Hospital, Oxford, United Kingdom; ^2^ CNC - Center for Neuroscience and Cell Biology, CIBB - Centre for Innovative Biomedicine and Biotechnology, University of Coimbra, Coimbra, Portugal; ^3^ Department of Neurobiology, Physiology and Behavior, College of Biological Sciences, University of California, Davis, Davis, CA, United States; ^4^ Department of Physiology and Membrane Biology, School of Medicine, University of California, Davis, Davis, CA, United States; ^5^ Institute of Metabolism and Systems Research (IMSR), University of Birmingham, Edgbaston, United Kingdom; ^6^ School of Life and Health Sciences, University of Roehampton, London, United Kingdom

**Keywords:** diabetes, islets, inter-cellular communication, connectivity, network, subpopulations, cilia

Islets of Langerhans are spherical clusters of endocrine cells embedded in the pancreatic parenchyma. These micro-organs are essential for the regulation of blood glucose metabolism through the release of insulin and glucagon from beta and alpha cells, respectively. The structural configuration of the islets confers a high degree of homotypic and heterotypic cell-cell contact which facilitates communication between the different cells. This sets the structural/functional basis for rapid and precise islet hormonal responses to systemic metabolic changes.

How do the pancreatic islets develop into this well-organized structure that is fit for their coordinated secretory response? In this Research Topic, Waters and Blum provide an overview of potential novel roles for axon guidance molecules in the formation and regulation of islet structure. Key players such as Slit-Robo, Semaphorin-Neuropilin, Ephrin-Eph and Netrins are considered in the context of islet morphogenesis as providers for chemotactic signalling cues influencing progenitor cell migration and, thus, islet architecture. Furthermore, these molecules are equally important in adult islet function and the implications for their altered functionalities in type 2 diabetes (T2D) are highlighted.

It is becoming evident that not all the beta cells are the same. The heterogeneous beta cell populations are electrically or chemically coupled, forming an insulin-releasing ‘syncytium’, responding to external stimuli (1, 2). The emergence of concepts such as subpopulations, hubs and first responder cells within subpopulations have potential functional implications in health and disease (3). Furthermore, it opens new avenues for the study of network connectivity within and between subpopulations across the islet using systems biology and bioinformatics. In that context, Stožer et al. provides an accessible and comprehensive review, presenting basic principles of network science and evaluating advanced methodologies to construct sophisticated simulations of islet networks based on multicellular imaging techniques. One such approach is exemplified by the original research by Postić et al., combining Ca^2+^ imaging in pancreatic tissue slices and network analysis to demonstrate the impact of pH on beta-cell connectivity. Current network models rely heavily on Ca^2+^ imaging data, but, as indicated by Stožer et al., there is scope for promising future developments such as multilayer networks, incorporating additional physiological factors in the computational analysis.

The concept of distinct islet subpopulations, whether that be beta or non-beta cell groups, is a recurrent theme throughout the collection of this special issue, including cell populations with mixed identity. Jin and Korol discuss in their mini-review of gamma-aminobutyric acid (GABA) signalling in human islets, the detection of insulin and glucagon co-expressing cells in tissue from T2D donors. They suggest that these subpopulations may be particularly amenable to modification by components of the GABA system based on observed differential GABA_A_ receptor channel activity in these groups.

Holter et al. similarly reports of transcriptional and functional heterogeneity of islet alpha cells, thus demonstrating that alpha cells exert important roles during the prandial phase that extend well beyond their primary counterregulatory role. This is linked to the production of GLP-1 by alpha cells under stress-related metabolic conditions, combined with the notion that islet-derived glucagon, like GLP-1, can activate GLP-1 receptors on beta cells to enhance insulin release. Subpopulations are thus identified based on cell-specific expression of glucagon, GLP-1 or both and are possibly associated with differences in proliferative capacity and state of cell maturity. The authors suggest that increased GLP-1 production by alpha cells may be an adaptive response to beta cell dysfunction and can promote beta cell proliferation, survival, insulin release and possibly cell-type conversion. In turn, this links to therapeutic potential in T2D and the implications for the current pharmacological targeting of GLP-1/GLP-1R are further evaluated.

The question of islet heterogeneity is extended to type 1 diabetes (T1D) by Brawerman et al. They previously identified a subgroup of beta cells in T1D donor islets characterized by a senescent phenotype associated with permanent growth arrest (4). In the current study they further investigate whether the same holds true for alpha cells, as dysfunctional glucagon secretion is a hallmark of T1D. Their analysis of islets from female NOD mice found distinct alpha-cell subgroups, that were primarily linked to differences in metabolism and immune responses, not to specific markers of senescence. This was corroborated by analysis of published RNA-sequence data as well as staining of alpha cells from human T1D donors. Interestingly, one small subgroup was characterized by both glucagon and somatostatin expression, suggesting altered cell identity as reported above by Jin and Korol. However, this subgroup did not become more prominent with the development of T1D.

Having highlighted islet heterogeneity, it becomes even more pertinent to explore the underlying mechanisms for the co-ordinated response of the whole islet. There remain unanswered questions, but one promising emerging factor is the primary cilia as explored by Pablos et al. and Cho and Hughes, respectively. These rod-like organelles are located at the surface of beta, alpha and delta cells as well as in the exocrine pancreas; they convey signals within and between adjacent cells and differentially express receptors, channels and signalling molecules involved in islet function. Pablos et al. provides an overview of cilia structure, formation and signal transduction in the context of islet function in health and disease and highlights how the study of ciliopathies have provided insights into their functional targets in islets and in the development of diabetes. This is further developed by Cho and Hughes, who, with current experimental models as springboard, discuss the scope of future research directions. The direct involvement of cilia in cell-intrinsic pathways modulating hormone release is considered as well as the potential for islet crosstalk between different cell types and indeed bi-directional signalling *via* cilia for example involving the axon guidance molecules Ephrin-Eph as highlighted above by Waters and Blum. There are suggestions of exciting avenues of research to pursue as much of the data on the involvement of cilia in islet cell function has been derived from model organisms; data from human islets is much needed in the quest to understand islet function for the management of diabetes.

The aim of this Research Topic was to identify recent advances in our knowledge of how the overall functional capacity of the pancreatic islets is regulated *via* coordinated intercellular communication between their different components. We have indeed explored islet cell heterogeneity and cross talk but, as indicated by the authors, there are still much to learn and exciting research directions to pursue to further increase our understanding of the highly coordinated release of hormones from the islet.

## Author contributions

AH-E drafted the editorial. QZ, MH and GX critically revised and edited the manuscript and approved the final version.

## References

[1] Fernandez J Valdeolmillos M . Synchronous glucose-dependent [Ca(2+)](i) oscillations in mouse pancreatic islets of langerhans recorded *in vivo* . FEBS Lett (2000) 477(1-2):33–6. doi: 10.1016/s0014-5793(00)01631-8 10899306

[2] Weitz J Menegaz D Caicedo A . Deciphering the complex communication networks that orchestrate pancreatic islet function. Diabetes (2021) 70(1):17–26. doi: 10.2337/dbi19-0033 33355306 PMC7881851

[3] Johnston NR Mitchell RK Haythorne E Pessoa RP Semplici F Ferrer J . Beta cell hubs dictate pancreatic islet responses to glucose. Cell Metab (2016) 24(3):389–401. doi: 10.1016/j.cmet.2016.06.020 27452146 PMC5031557

[4] Thompson PJ Shah A Ntranos V Van Gool F Atkinson M Bhushan A . Targeted elimination of senescent beta cells prevents type 1 diabetes. Cell Metab (2019) 29:1045–60. doi: 10.1016/j.cmet.2019.01.021 30799288

